# Apixaban returned to CVS Caremark formulary after outcry

**DOI:** 10.1002/rth2.12774

**Published:** 2022-07-26

**Authors:** Beth Waldron

**Affiliations:** ^1^ Independent Patient Advocate Chapel Hill North Carolina USA

In follow‐up to my article “Nonmedical switching of anticoagulants: The patient impact when formulary exclusions limit drug choice” published March 15, 2022, I am pleased to share that pharmacy benefits manager CVS Caremark has restored apixaban to its national commercial formulary after a 6‐month absence, effective July 1, 2022. The reversal came after an unprecedented outpouring of opposition from patients, physicians, and medical nonprofits.

The reach of my *RPTH* article far exceeded my expectations, extending well beyond the thrombosis community. I heard from one rheumatology practice who began including it in their prior authorization requests for rituximab as documentation of the patient impact of nonmedical drug switching in general. Although there has been research on the administrative burden of nonmedical switching, prior authorizations and other insurance practices increasingly used as barriers to care, there has been very little study on the patient perspective of these practices, and I suggest this is an area worthy of future formal study.

Since publication, there have been reports of adverse events resulting from this nonmedical switch of anticoagulants.^1^, ^2^, ^3^ I highly encourage clinicians with knowledge of such events to consider publishing a case report. Given the narrow time window of this forced medication change, there is a unique opportunity to be able to clearly demonstrate causality. Having adverse event cases published in the citable medical literature will help make the policy argument that such nonmedical switching practices should be banned in the future.

Additionally, I hope nonprofits and clinicians will advocate for anticoagulants to be classified as a protected drug class by the Centers for Medicare & Medicaid Services. Current policy requires Part D sponsors to include on their formularies all drugs in six categories: anticonvulsants, antidepressants, antineoplastics, antipsychotics, antiretrovirals, and immunosuppressants. What happens in Medicare tends to be taken up in the commercial insurance industry. Given the unique risks associated with anticoagulants, they absolutely should be included alongside these protected drugs so that their selection and management is only made by a knowledgeable clinician, not an insurance company executive.

I appreciate *RPTH* for providing a welcoming home for science and discourse among researchers, clinicians, AND patients! The reversal of the apixaban formulary drop by CVS Caremark is testament that rapid change is possible in the complex American health system when patients, clinicians, and nonprofits join forces together for collective action. It is, I suggest, an effective model for meeting future challenges.

## RELATIONSHIP DISCLOSURE

The authors have no conflicts of interest to disclose.
